# Supplementary figures and images for: Excalibur: A new ensemble method based on an optimal combination of aggregation tests for rare-variant association testing for sequencing data
Source: PLoS Comput Biol. 2023 Sep 14;19(9):e1011488. doi: 10.1371/journal.pcbi.1011488 (PMC10522036; doi:10.1371/journal.pcbi.1011488)

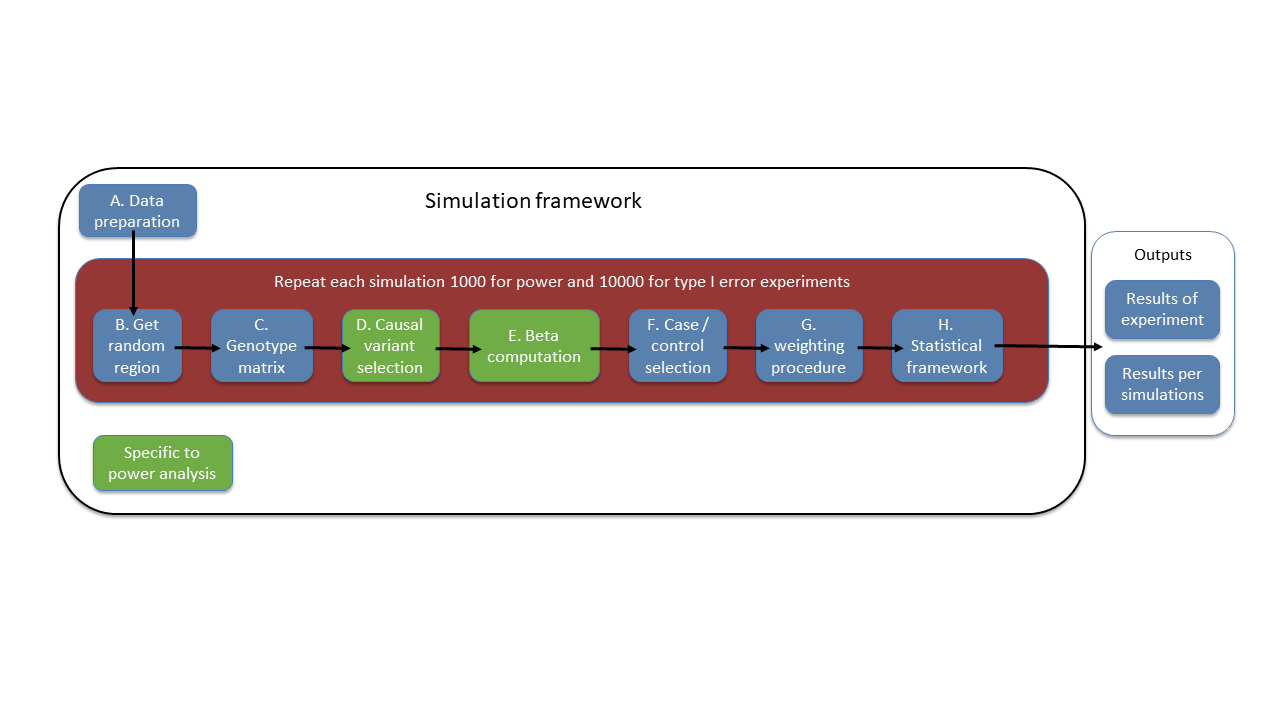

Supplement: S1 Fig — Schema of the code structure (independent modules represented in blue or green boxes) and data flow (black arrows) of our simulation framework. The green boxes represent steps that are specific to empirical power simulations. (PNG) [file pcbi.1011488.s001.png]

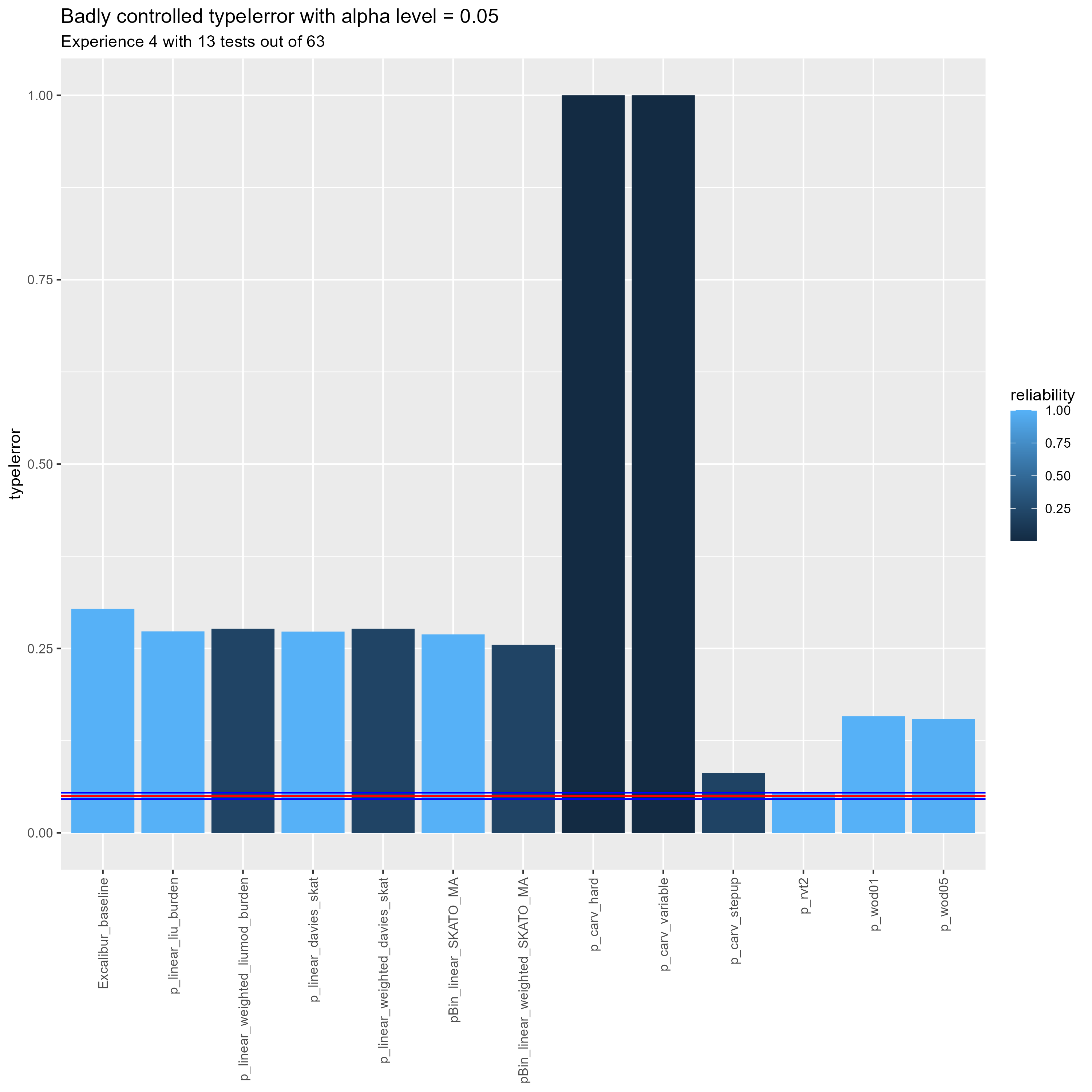

Supplement: S2 Fig — Methods (X axis) that had an inflated type I error for experiment ID n°4 (Table 1) and their type I error (Y axis) at nominal level α = 0.05 based on 10 000 replicates. The red line corresponds to α = 0.05 and blue lines correspond to 95% confidence interval. Confidence interval computed assuming that the number of false positives follows a binomial distribution with parameters 10,000 and 0.05. Each bar is colored given the reliability. (PNG) [file pcbi.1011488.s002.png]

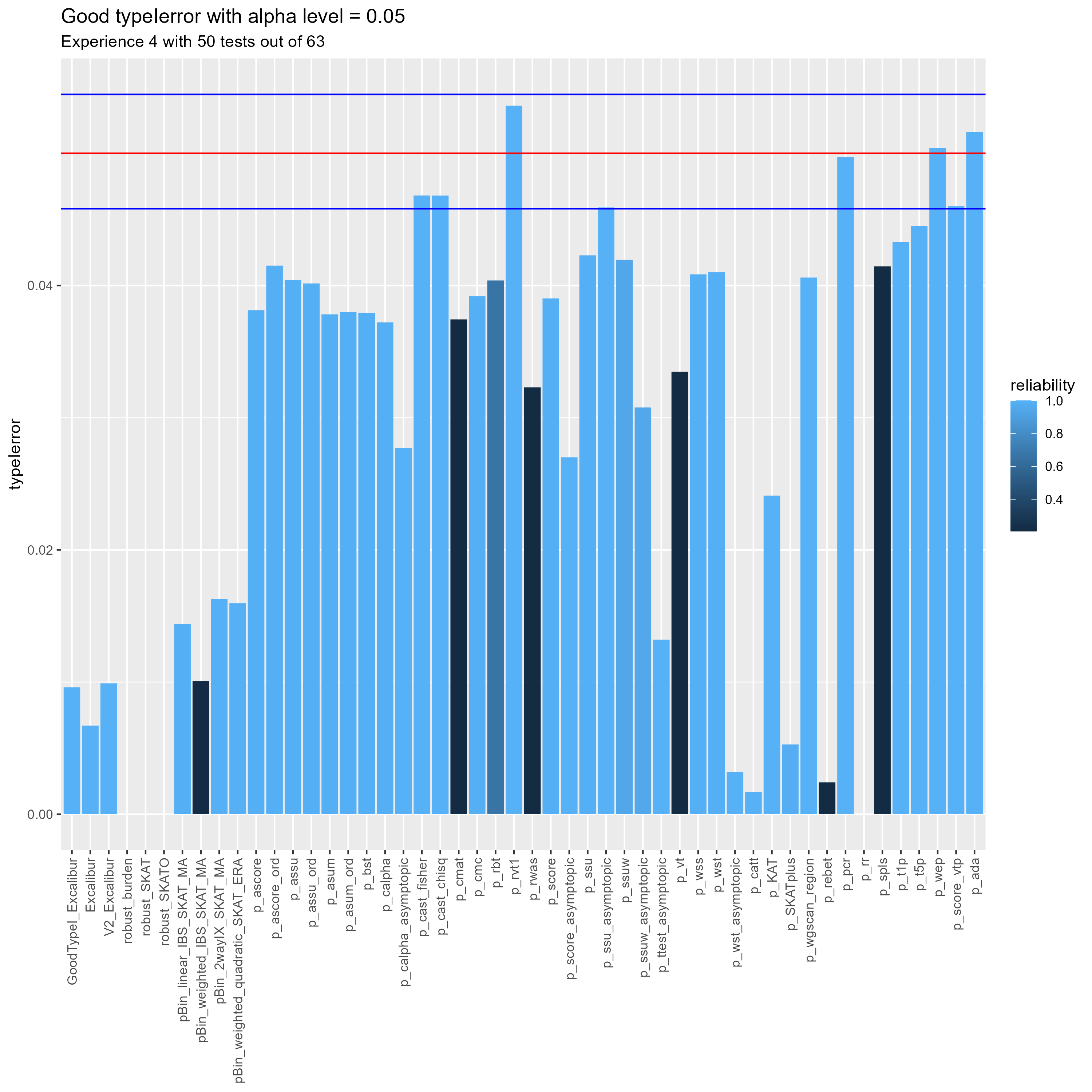

Supplement: S3 Fig — Methods (X axis) that have a good type I error for experiment ID n°4 (Table 1) and their type I error (Y axis) at nominal level α = 0.05, based on 10 000 replicates. The red line corresponds to α = 0.05 and blue lines correspond to 95% confidence interval. Confidence interval computed assuming that the number of false positives follows a binomial distribution with parameters 10,000 and 0.05. Each bar is colored given the reliability. (PNG) [file pcbi.1011488.s003.png]

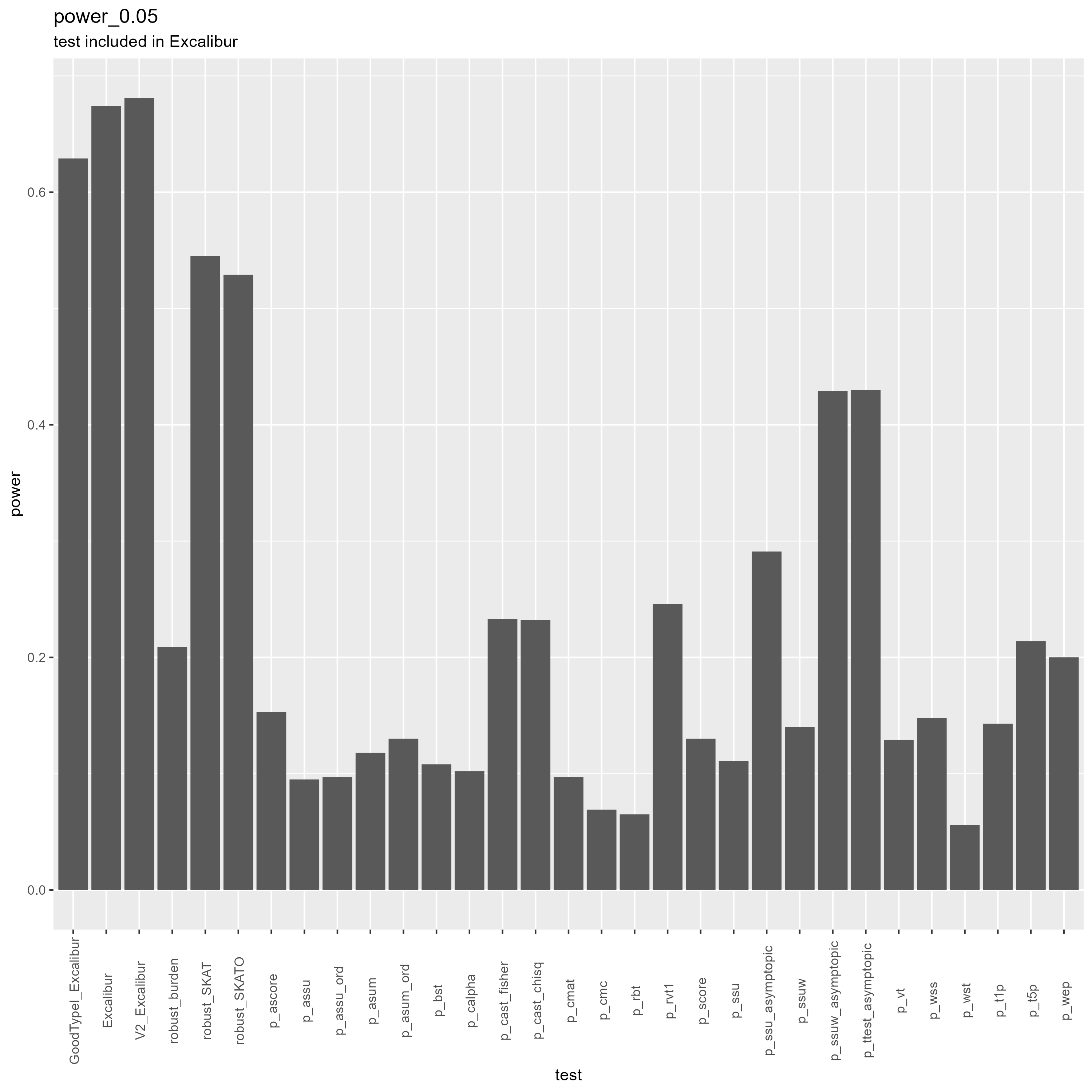

Supplement: S4 Fig — Plots for methods (X axis) having proportion of inflated type I error equal to zero (S4 Table) and their empirical power (Y axis) at nominal level α = 0.05 based on 1000 replicates for experiment ID n°9 (Table 1). (PNG) [file pcbi.1011488.s004.png]

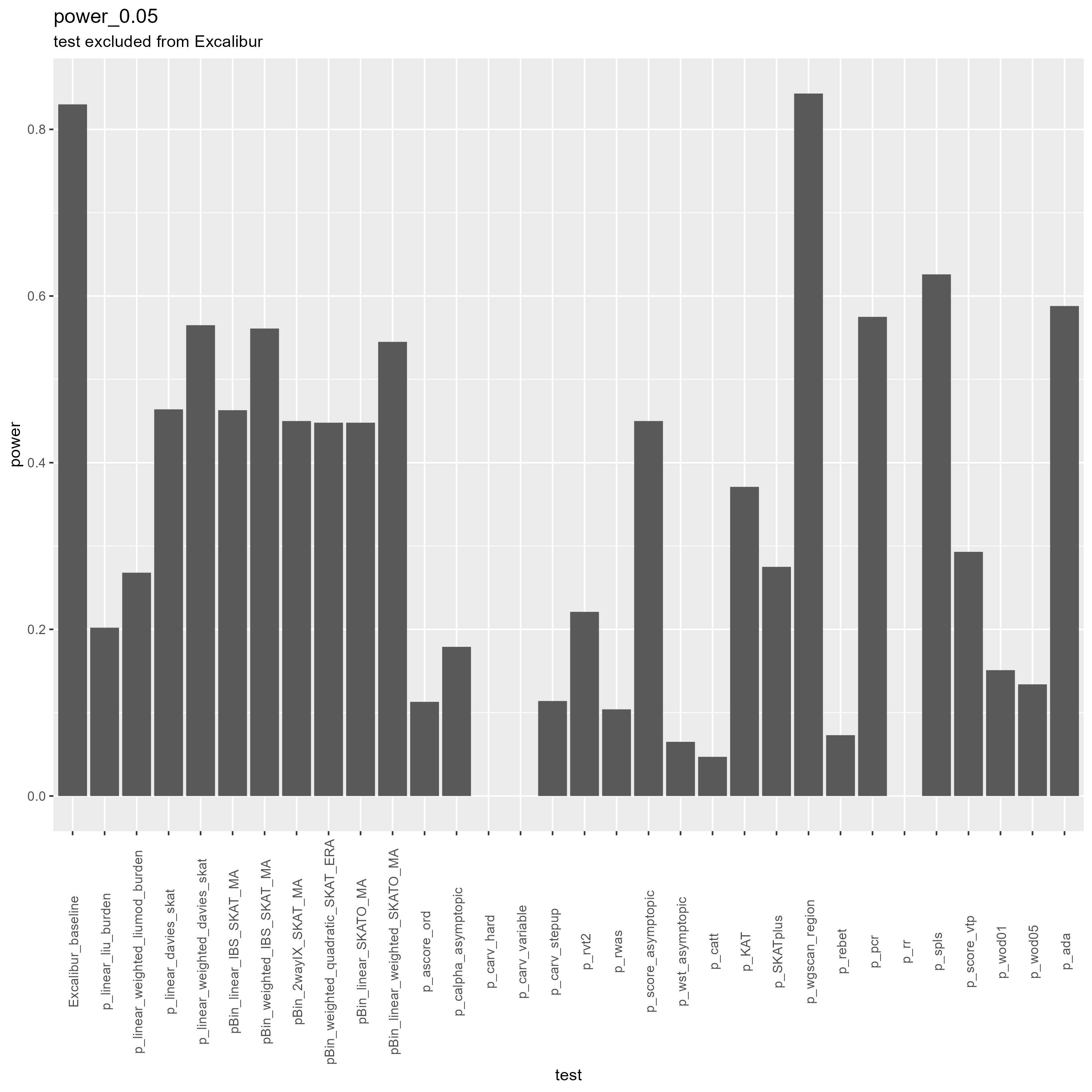

Supplement: S5 Fig — Plots for methods (X axis) with proportion of inflated type I error above zero (S4 Table) and their empirical power (Y axis) at nominal level α = 0.05, based on 1000 replicates for experiment ID n°9 (Table 1). (PNG) [file pcbi.1011488.s005.png]

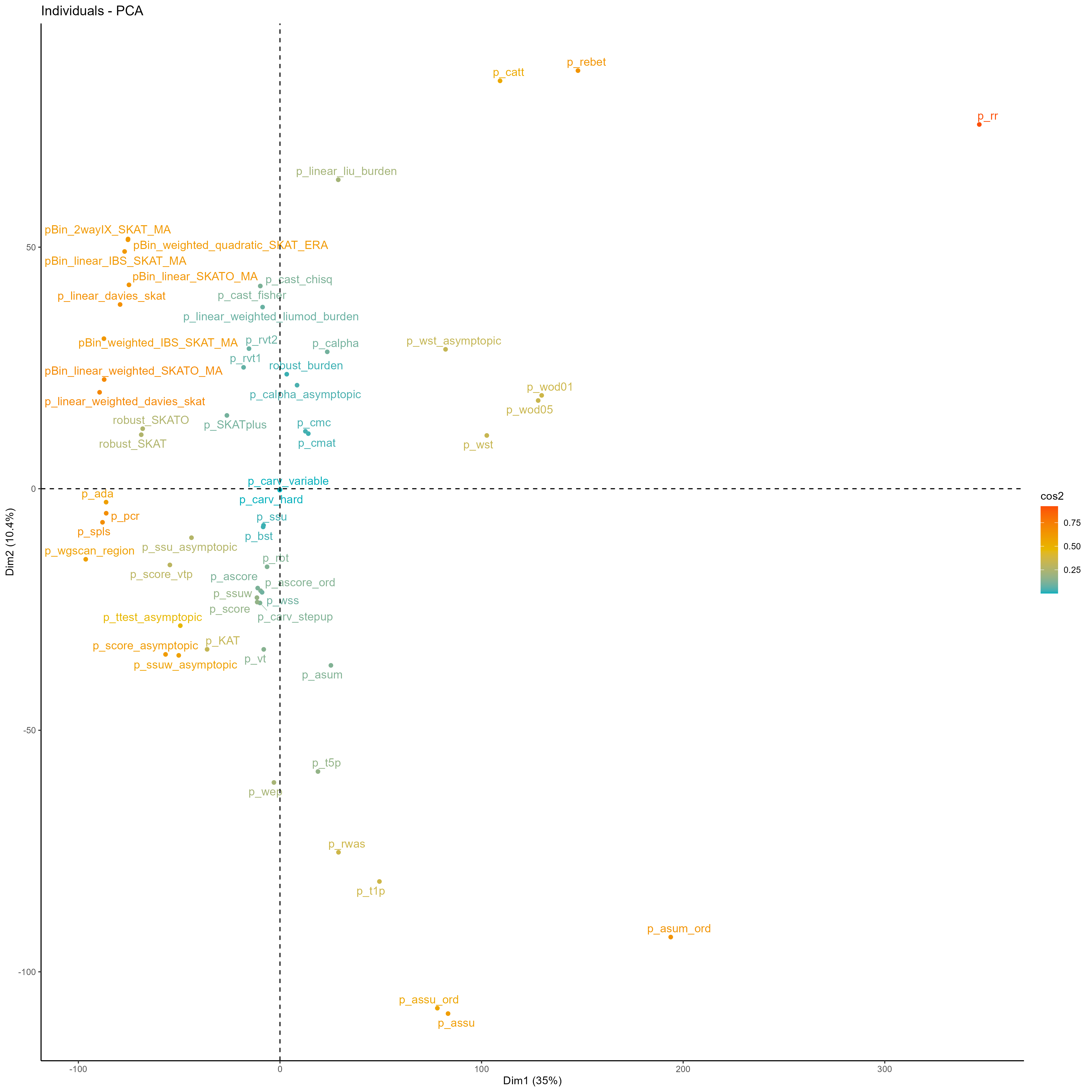

Supplement: S6 Fig — Plot of first principal component (X axis) and second principal component (Y axis) of 59 state-of-the-art methods colored by cos2: squared cosine values, indicate the contribution of each variable to a specific principal component. Higher cos2 values imply a stronger correlation between the variable and the principal component, indicating a better representation of the variable on the plot. The principal component analysis is based on 18,000 empirical power simulations for each test. (PNG) [file pcbi.1011488.s006.png]

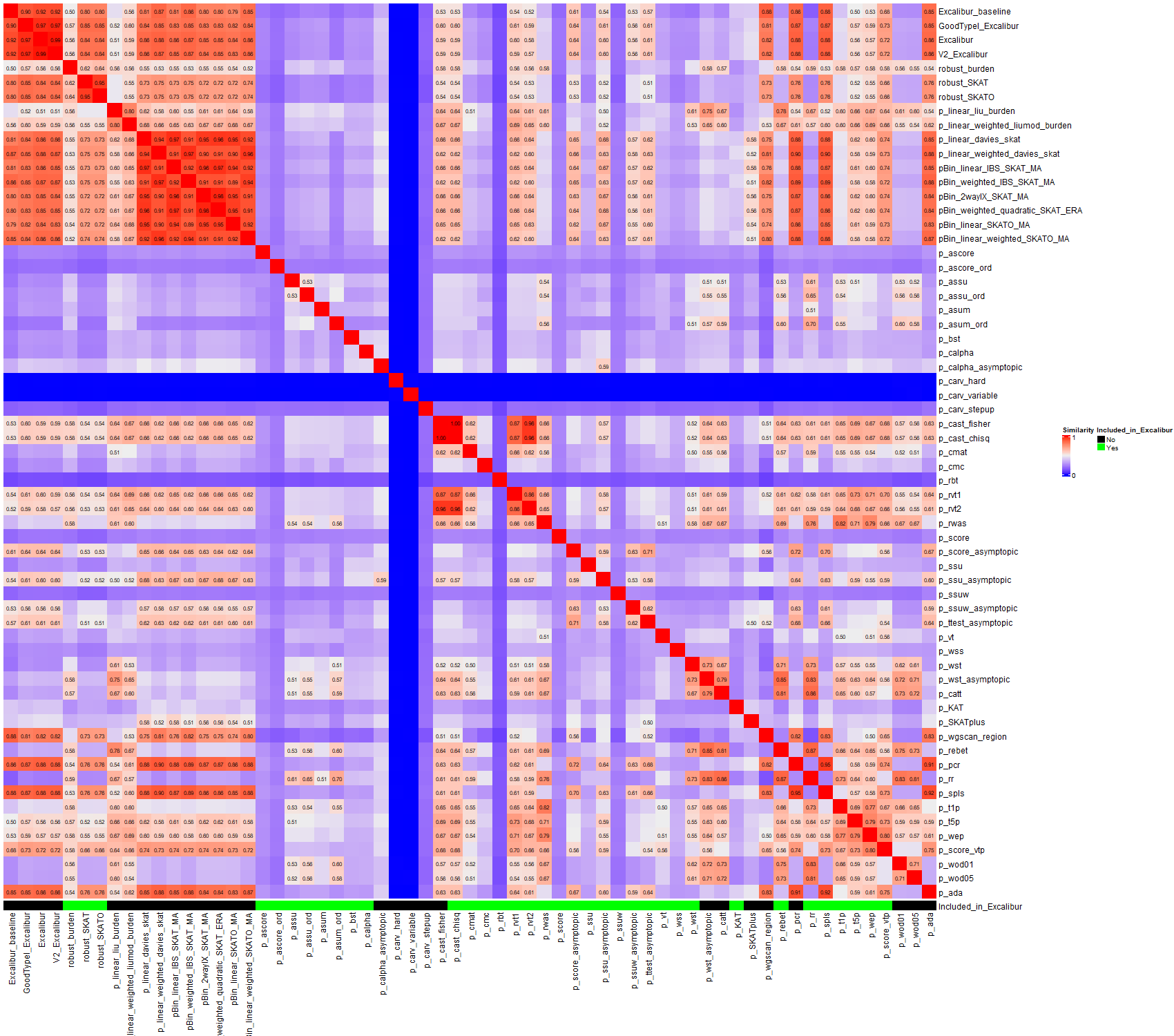

Supplement: S7 Fig — Heatmap of similarities, ranging from zero (in blue), to 1 (in red), of our 4 ensemble methods and 59 state-of-the-art methods (X and Y axis). Similarity is defined as the proportion of simulation where two tests give the same output (significant or non-significant) evaluated at nominal level α = 0.05 out of the 18,000 empirical power simulations. Only similarities above 0.5 are displayed. Green: test is included in Excalibur. Black: test is not included in Excalibur, or is one of our ensemble methods. (PNG) [file pcbi.1011488.s007.png]

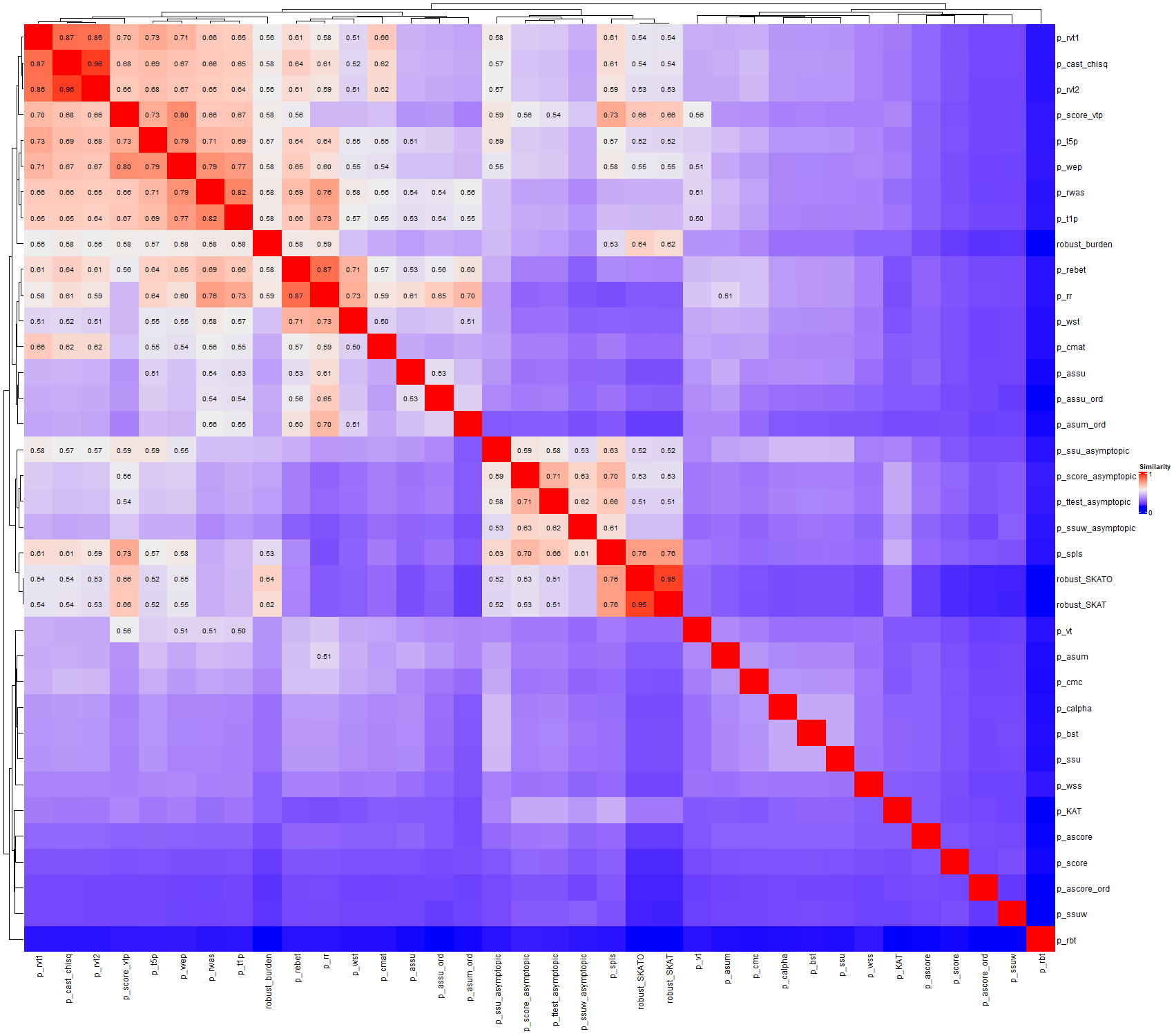

Supplement: S8 Fig — Hierarchical clustering of similarities, ranging from zero (in blue), to 1 (in red), of 36 state-of-the-art methods (X and Y axis) included in Excalibur. Similarity is defined as the proportion of simulation where two test give the same output (significant or non-significant) evaluated at nominal level α = 0.05 out of the 18,000 empirical power simulations. Only similarities above 0.5 are displayed. (PNG) [file pcbi.1011488.s008.png]

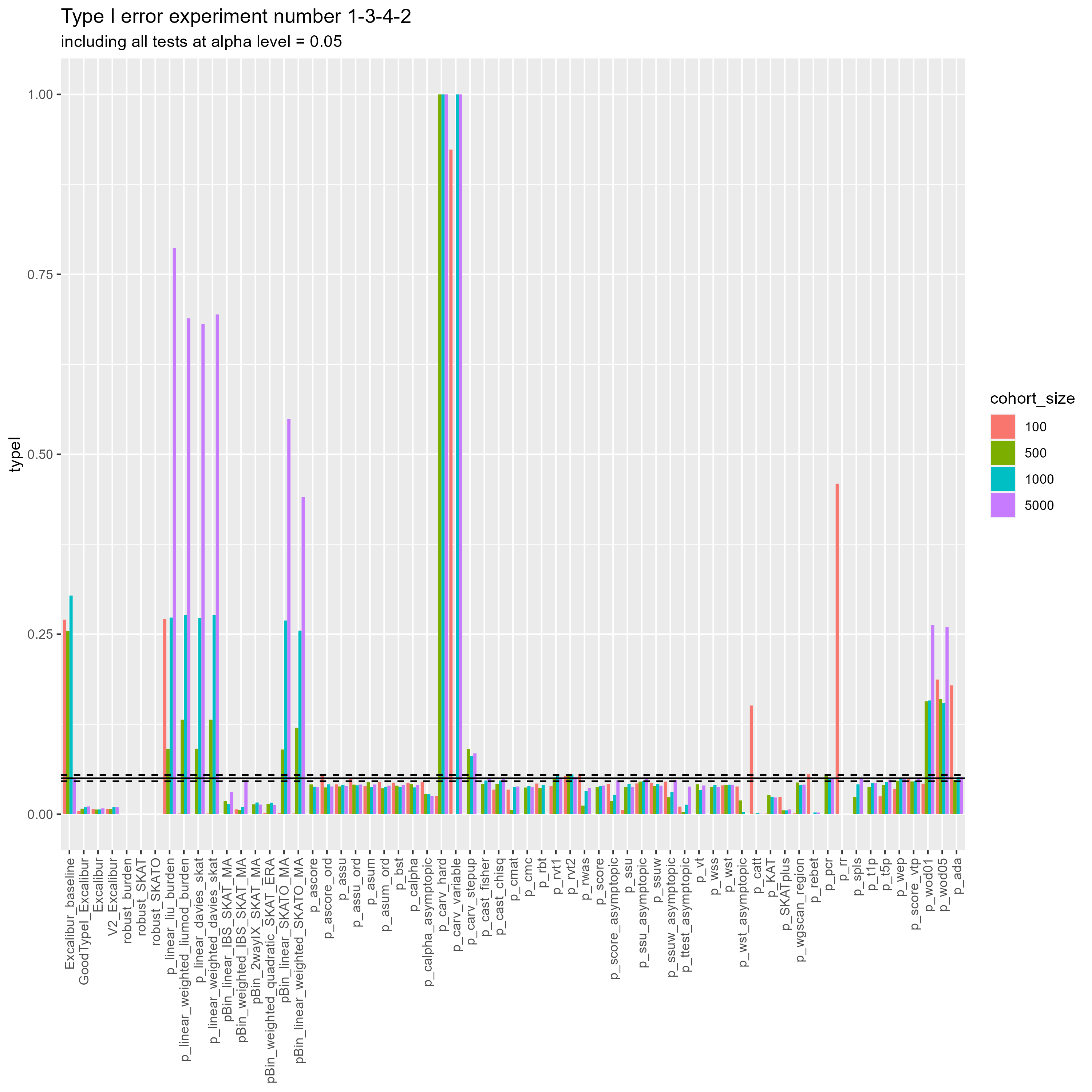

Supplement: S9 Fig — Plot for our 4 ensemble methods and 59 state-of-the-art methods (X axis) and their type I errors (Y axis) at nominal level α = 0.05 for experiment ID n°1 in red, n°2 in green, n°3 in blue and n°4 in magenta (Table 1). Type I error results based on 10 000 replicates for each experiment. The straight black line corresponds to α = 0.05 and dashed black lines correspond to 95% confidence interval. Confidence interval computed assuming that the number of false positives follows a binomial distribution with parameters 10,000 and 0.05. All experiments sare based on exact same parameters except for the cohort size and shows the impact of that parameter on the behavior of all aggregation tests analyzed. (PNG) [file pcbi.1011488.s009.png]

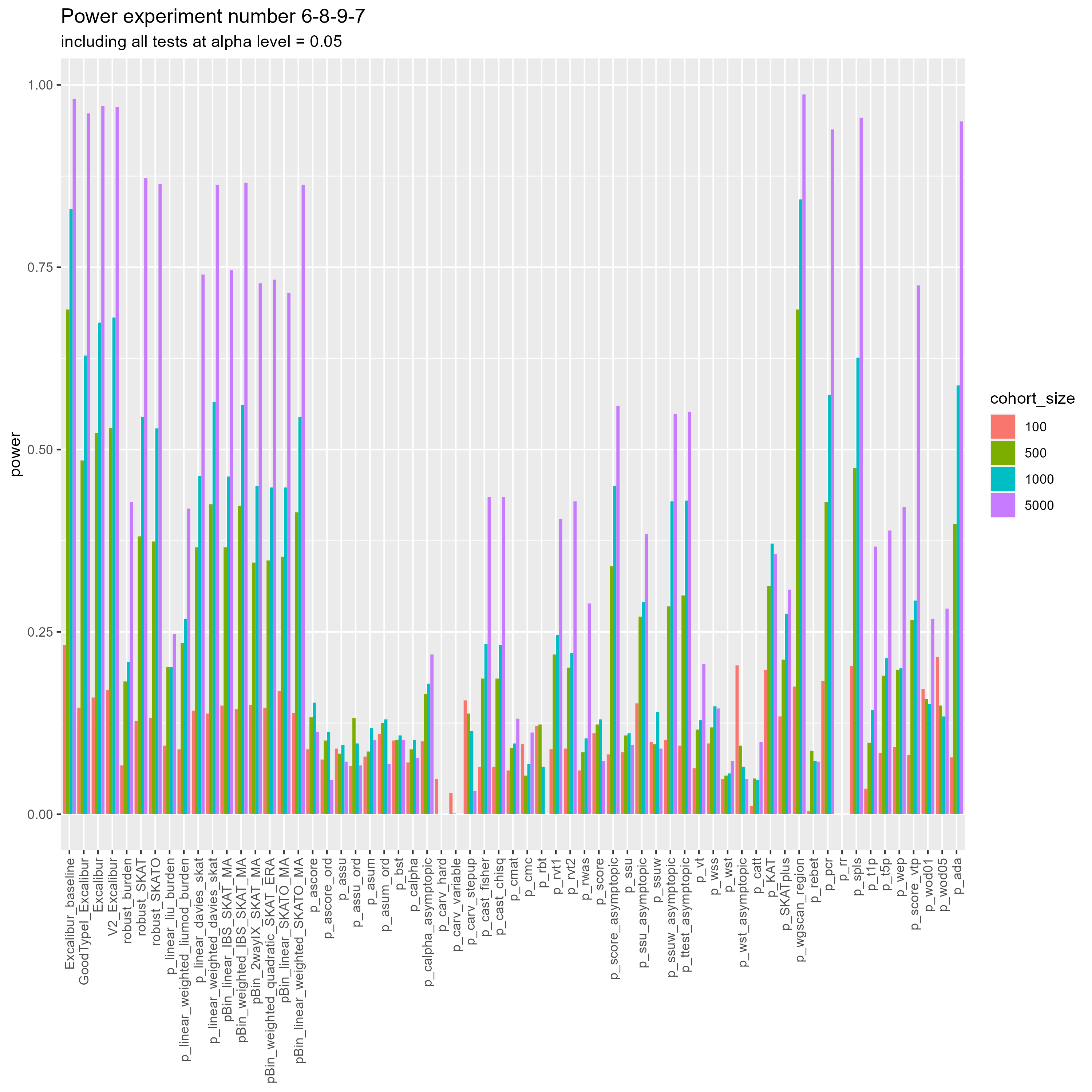

Supplement: S10 Fig — Plot for our 4 ensemble methods and 59 state-of-the-art methods (X axis) and their empirical power (Y axis) at nominal level α = 0.05 for experiment ID n°1 in red, n°2 in green, n°3 in blue and n°4 in magenta (Table 1). Empirical power results based on 1000 replicates for each experiment. All simulations were based on exact same parameters except for the cohort size and show the impact of that parameter on the behavior of all aggregation tests analyzed. (PNG) [file pcbi.1011488.s010.png]

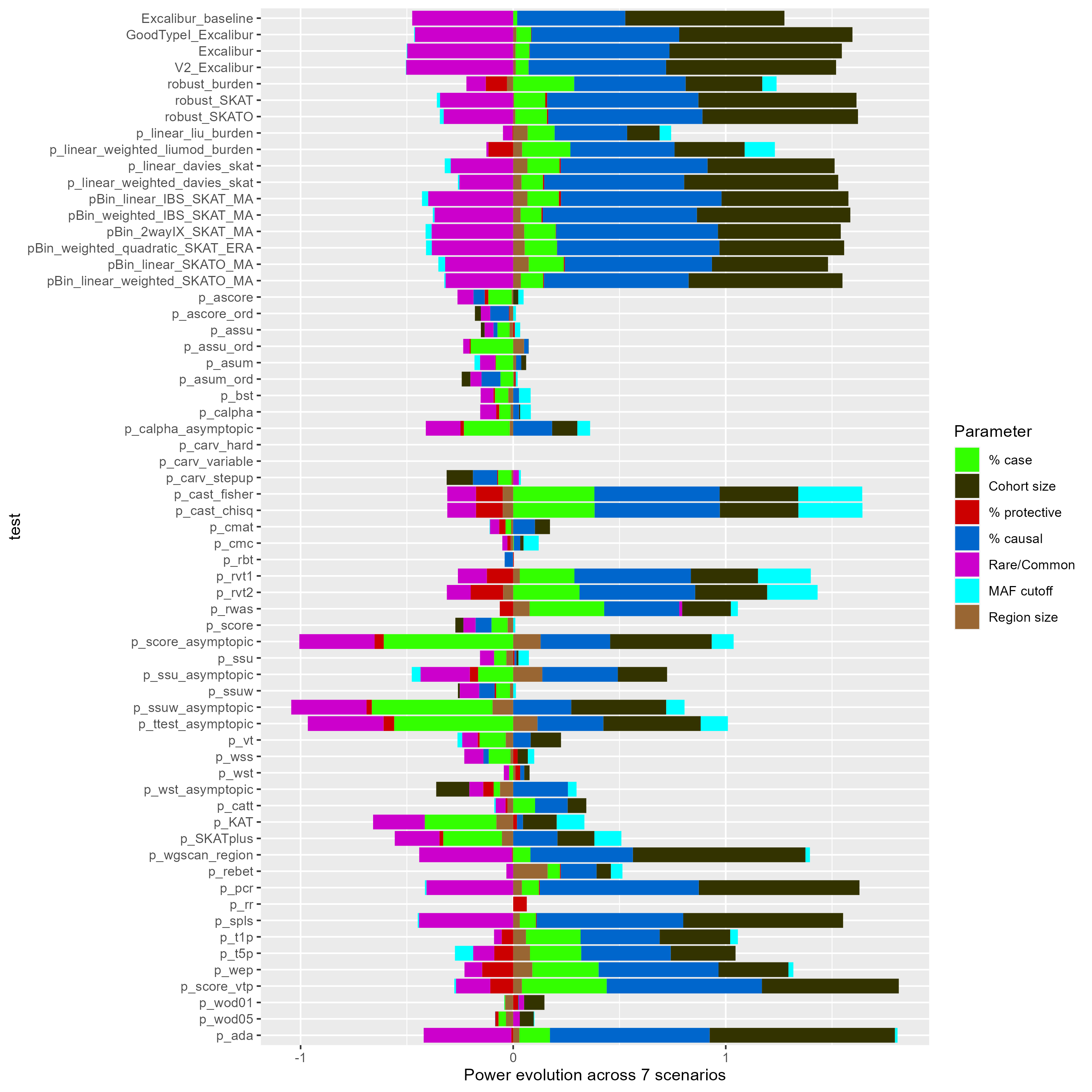

Supplement: S11 Fig — Plot for our 4 ensemble methods and 59 state-of-the-art methods (X axis) and their total empirical power evolution (Y axis) at nominal level α = 0.05 for seven scenarios (see colors), based on S10 Table. Green: evolution of power given the evolution of proportion of case in the cohort, based on empirical power ID n°14, n°11 and n°15 (Tables 1 and S5). Black: evolution of power given the evolution of cohort size, based on empirical power ID n°6, n°7, n°8 and n°9 (Tables 1 and S5). Red: evolution of power given the evolution of proportion of protective variants, based on empirical power ID n°11, n°12 and n°13 (Tables 1 and S5). Blue: evolution of power given the evolution of proportion of causal variants, based on empirical power ID n°2, n°3, n°4 and n°5 (Tables 1 and S5). Magenta: evolution of power given the inclusion of only rare variants versus rare and common variants, based on empiric al power ID n°18 and n°10 (Tables 1 and S5). Turquoise: evolution of power given the evolution of causal MAF cutoff, based on empirical power ID n°1 and n°11 (Tables 1 and S5). Brown: evolution of power given the evolution of region size, based on empirical power ID n°17 and n°16 (Tables 1 and S5). (PNG) [file pcbi.1011488.s011.png]

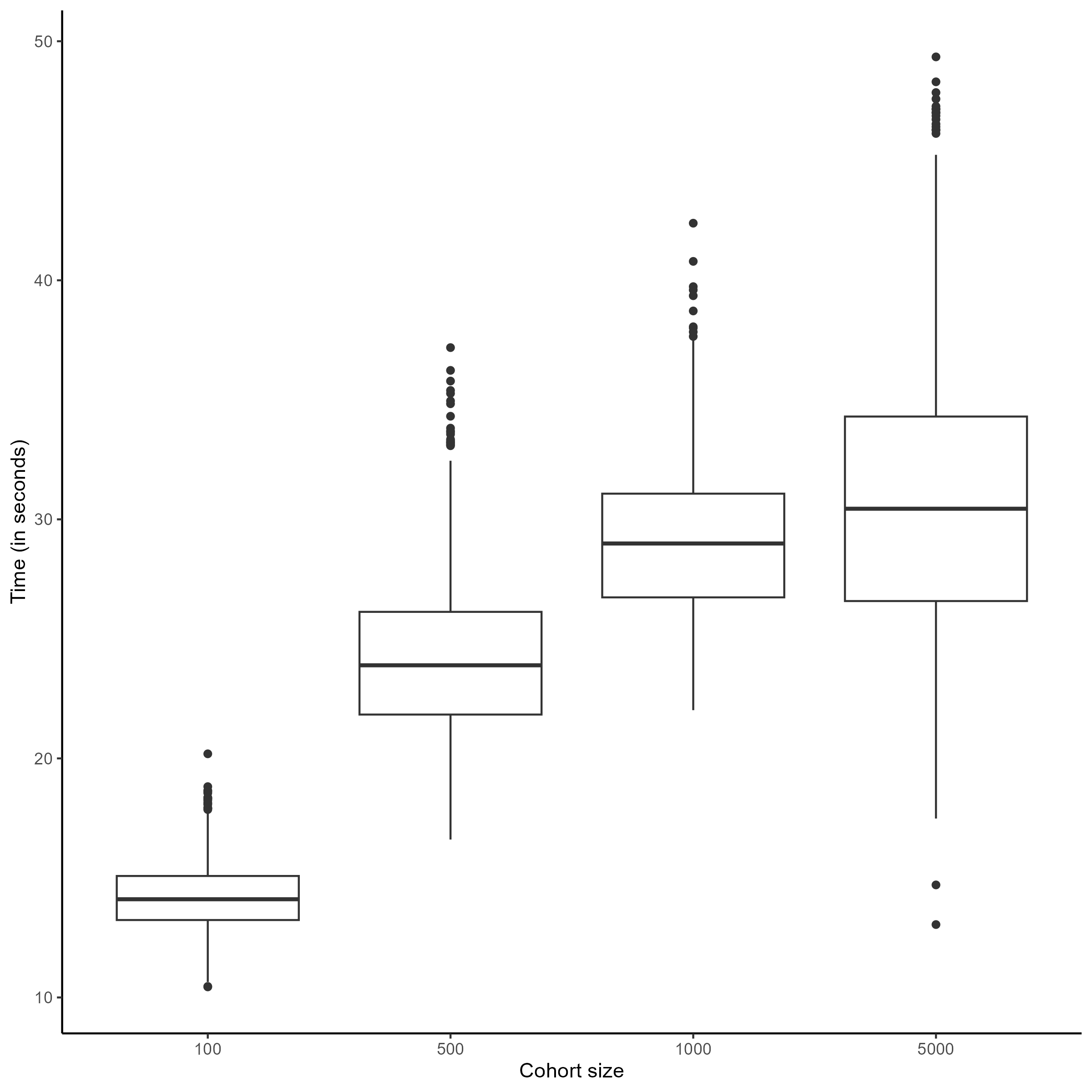

Supplement: S12 Fig — Boxplot illustrating the computational time (in seconds) required by the Excalibur method across varying cohort sizes. The x-axis represents the cohort size, while the y-axis denotes the computational time in seconds. The distribution of computational time is visualized using boxplot, providing insights into the method’s efficiency as cohort size changes. (PNG) [file pcbi.1011488.s012.png]
