# Supplementary material for: Excalibur: A new ensemble method based on an optimal combination of aggregation tests for rare-variant association testing for sequencing data
Source: PLoS Comput Biol. 2023 Sep 14;19(9):e1011488. doi: 10.1371/journal.pcbi.1011488 (PMC10522036; doi:10.1371/journal.pcbi.1011488)
Supplement: Attachment — Submitted filename: Review_forauthors.docx [file pcbi.1011488.s026.docx]

The authors present a comparison of multiple aggregation tests for rare variant analysis in terms of type I error and power levels. To this end, extensive simulations are performed by varying several parameters including the cohort size and the proportion of causal variants. The authors show that only some of the methods maintain good type I error levels in all the scenarios at three different alpha levels. Power values should therefore be interpreted in light of the type I error values to make sure that significant signals do not correspond to false positives. In addition, the authors propose a new method, Excalibur, to combine the aggregation tests that maintain good type I errors by considering the corrected minimal p-value. They show that Excalibur maintain good type I error levels and presents the highest power across multiple scenarios. Overall, there is a large effort in the simulated scenarios to characterize multiple aggregation tests implemented in R. While some results were already reported in the literature, this study offers useful insights in how the actual implemented tests behave. Nevertheless, I am less convinced about the new proposed method which considers only the minimal p-value of multiple tests rather than combining them. In practice, it is of common usage to try multiple aggregation tests to consider multiple hypotheses and then correct the p-values and I am therefore unsure about the novelty of this approach. Of particular concern is the use of multiple aggregation tests that seem to be correlated and would therefore negatively impact the power of the combined method, as well as the computation time needed for Excalibur to run. In addition, the add-on of Excalibur could be to propose an easy-to-use package to perform all the tests at once, but it seems that only one function is available on Github without a comprehensive documentation and without being integrated in a proper package taking care of all the dependencies to be installed. Finally, the overall manuscript needs more clarification of several aspects which are listed in details below.

# Major comments

The four new ensemble methods should be presented much earlier in the manuscript and should be placed in the methods section so that the manuscript is easier to follow.

Overall, it seems that the correlation between the different aggregation tests is not directly considered in the new ensemble methods. For example, in Excalibur, p_assu/p_assu_ord as well as p_ssu/ p_ssu_asymptotic are selected. These represent alternatives of the same tests and are expected to give very similar p-values. I would expect that this will reduce the power due to the multiple testing correction.

Can the authors better explain why Excalibur is the ensemble method that is recommended? How do the authors interpret the opposite trends observed for the four ensemble methods in Figure 3?

The authors promote Excalibur especially for the analysis of WGS and WES data where different genetic architecture can be observed depending on the genomic area. Most WGS and WES analyses define significant regions using a genome-wide significance threshold (usually 2.5e-6 for the 20,000 genes in the genome in a WES analysis). It would therefore be useful to have type I error and power estimations at genome-wide significance thresholds, especially because it seems on Figure 1 that inflation patterns depend on the threshold considered.

One thing that is not mentioned in the manuscript is the computation time needed to run Excalibur. Even if tests with low computation time were selected at the beginning, Excalibur needs to run 33 tests. In addition, the sample sizes used in the simulations are quite small, so it is hard to get an idea of how efficient it is to use Excalibur on larger datasets and when analyzing WES or WGS data. It would be good to add a section in the manuscript investigating this question.

Can the authors explain why the type I error is estimated as the number of significant signals divided by the number of non-significant signals and not by the number of replicates for which a p-value was estimated (line 245)?

# Minor comments

I am wondering whether it would be possible to perform some PCA or clustering approach on the power estimations or the power evolutions. This would enable to see if some of the aggregation tests group together and which parameters drive these patterns (refers among others to lines 480-481).

Regarding the simulations, in the scenarios involving the analysis of both rare and common variants, can the common variants be selected as causal ones? If not, it seems that these scenarios meet the scenario of increasing the percentage of non-causal variants in the region. In addition, why are different weights given to the variants depending on the percentage of causal variants (line 294)? In that case, two parameters are varied at the same time.

l. 55-56: the authors claim that Excalibur ‘*automates the test selection depending on the genetic region to be explored’*. This is quite unclear and not really developed in the rest of the manuscript.

In addition to the proportion of inflated, it would be useful to have the proportion of conservative tests as it seems to be the case for Excalibur.

l. 284: Please clarify the sentence around the ‘*mathematical models*’. ID9 represents a scenario with a larger region than scenarios 3 and 8: can the authors explain why no results are obtained?

Comments on the supplementary tables:

- Table S2 would be useful as a main table as a lot of results refer to it.
- Could the authors give more digits in Table S4? This would be useful for the last significance threshold but also for a threshold of 0.01 when results are borderline (as for WGScan).
- Please define more precisely what is in Table S10 and how the evolution is computed.

Could the authors clarify the following points:

- l. 145: what does ‘out-of-the-box’ mean?
- l. 153: If a function has a wider range of computation time, for example going from 16 to 24 seconds, it will be privileged over others with a lower range but a lower computation time, for example going from 6 to 8seconds. Should the mean computation time also be considered?
- l. 163: what are the three scenarios considered? The three cohort sizes?
- l. 194: Why the number of causal variants need to be respected for the simulations under the null?
- l. 254: the proportion should be divided by the number of scenarios for which a type I error was estimated. Similarly in line 298, it should be divided by the number of experiments that gave a p-value.
- l. 421: Why can’t this be extended to other alpha levels?
- l. 451: It sound like the type I error is computed again, is it really the case or simply the minimal type I error?
- l. 465: How is the combined rank defined?
- Figures S2 and S3: Why p_rvt2 is in the badly corrected and not the goodly corrected panel?

The manuscript should be corrected for the following typos if I am not mistaking:

- l. 227: it is stated that this model is used to generate data under the null hypothesis. I think this is a mistake, especially as the null model is already stated line 199.
- l. 240: Table S4 is cited before Table S3
- l. 261: I think the authors refer to Fig S2 and S3 and not S1 and S2
- l. 273: typo error for the upper bound of the confidence interval for alpha = 0.01
- l. 287: there is an extra ‘s’ in Tables S13
- Table S11 is not cited in the text and table S12 is not really described.
