# Supplementary material for: Excalibur: A new ensemble method based on an optimal combination of aggregation tests for rare-variant association testing for sequencing data
Source: PLoS Comput Biol. 2023 Sep 14;19(9):e1011488. doi: 10.1371/journal.pcbi.1011488 (PMC10522036; doi:10.1371/journal.pcbi.1011488)
Supplement: Attachment — Submitted filename: response_to_reviewers.docx [file pcbi.1011488.s027.docx]

# Reviewer 1

## Major comments

- The four new ensemble methods should be presented much earlier in the manuscript and should be placed in the methods section so that the manuscript is easier to follow.
- We have changed the manuscript, added the description of the four new ensemble methods in the methods section and put that section right after the preliminary test selection.
- Overall, it seems that the correlation between the different aggregation tests is not directly considered in the new ensemble methods. For example, in Excalibur, p_assu/p_assu_ord as well as p_ssu/ p_ssu_asymptotic are selected. These represent alternatives of the same tests and are expected to give very similar p-values. I would expect that this will reduce the power due to the multiple testing correction.
- To evaluate test similarities, we compared all p-values from each test at nominal alpha 0.05 to establish their similarities (see new Figures S7 and S8). The highest similarity was achieved by p_cast_chisq/p_rvt2. These tests agreed (both significant or both not significant) in 96% of the simulations. The algorithm to construct Excalibur takes a test into account only when it is the only significant test for a simulation, which helps counterbalance the loss of power caused by multiple testing correction. A test will not be added to the ensemble method if it results in a decrease in power. Surprisingly, p_assu/p_assu_ord and p_ssu/ p_ssu_asymptotic only have a similarity of 53% and 46%, respectively.
- Can the authors better explain why Excalibur is the ensemble method that is recommended? How do the authors interpret the opposite trends observed for the four ensemble methods in Figure 3?
- Overall, Excalibur is recommended because it is the method that increases power across all experiments while controlling type I error (see Figure 2). The goal was to construct an ensemble method robust in a wider range of scenarios. Figure 3 should be interpreted looking at Table S12. For example, when including more protective variants, the power of GoodTypeI_Excalibur slightly increases (0.017), while it decreases in Excalibur (-0.004). This can be explained by the fact that the latter includes more Burden tests.
- The authors promote Excalibur especially for the analysis of WGS and WES data where different genetic architecture can be observed depending on the genomic area. Most WGS and WES analyses define significant regions using a genome-wide significance threshold (usually 2.5e-6 for the 20,000 genes in the genome in a WES analysis). It would therefore be useful to have type I error and power estimations at genome-wide significance thresholds, especially because it seems on Figure 1 that inflation patterns depend on the threshold considered.
- We completely agree with the reviewer. We parallelized the simulation framework (new code is available at <https://github.com/dduv-ddit/excalibur_simulations>). However, due to computational time concerns, we restricted our simulations to 10 000 repeats for each type I error experiment. In order to evaluate at a genome-wide significance, 10^7 simulations per experiment would be required to expect at least 10^7 * (2.5*10^(-6)) = 25 false positive results. With the computational power available at the institute, it would require a year to perform that many simulations (2 nodes with 16 cores (AMD EPYC MILAN 7313 Processor at 3GHz) and 100 Gb or RAM (DDR4 at 3200 MHz)). Moreover, most aggregation articles do not evaluate type I error at such threshold. Some papers implement different procedures to evaluate such thresholds (<https://doi.org/10.1093/biostatistics/kxs014>, 10.1016/j.ajhg.2011.05.029, 10.1016/j.ajhg.2012.06.007, 10.1016/j.ajhg.2019.11.012). However, they are not described well enough to be reproduced and we do not have access to their supplementary data or code used to perform such simulations. We contacted the authors, without success.
- One thing that is not mentioned in the manuscript is the computation time needed to run Excalibur. Even if tests with low computation time were selected at the beginning, Excalibur needs to run 33 tests. In addition, the sample sizes used in the simulations are quite small, so it is hard to get an idea of how efficient it is to use Excalibur on larger datasets and when analyzing WES or WGS data. It would be good to add a section in the manuscript investigating this question.
- In order to answer the question, we changed our experiments with a cohort size of 200 to 5000. Now, the four cohort sizes being investigated are 100, 500, 1000 and 5000. We also added a section to discuss the computational time of each method in the revised manuscript.
- Can the authors explain why the type I error is estimated as the number of significant signals divided by the number of non-significant signals and not by the number of replicates for which a p-value was estimated (line 245)?
- This is based on the fact that sometimes some tests do not manage to return a p-value (see reliability). Therefore, using the number of significant signals divided by the number of replicates, for example for the test, returned 10 / 10,000 = 0.0010, but if reliability was 0.9, we would only have 10 / 9,000 = 0.0011.

## Minor comments

- I am wondering whether it would be possible to perform some PCA or clustering approach on the power estimations or the power evolutions. This would enable to see if some of the aggregation tests group together and which parameters drive these patterns (refers among others to lines 480-481).
- We performed an additional PCA analysis based on all 18,000 empirical power simulation results for each test, and changed the manuscript accordingly (see new Figure S6).
- Regarding the simulations, in the scenarios involving the analysis of both rare and common variants, can the common variants be selected as causal ones? If not, it seems that these scenarios meet the scenario of increasing the percentage of non-causal variants in the region. In addition, why are different weights given to the variants depending on the percentage of causal variants (line 294)? In that case, two parameters are varied at the same time.
- Yes, common variants can be selected as causal ones. The weights follow the strategies used. For the second part of the question, I think the reviewer refers to line 230-235 of the original manuscript (line 286 - 292 of the revised manuscript with track changes). We try to simplify the manuscript, but this is directly taken from the literature cited in the manuscript.
- l. 55-56: the authors claim that Excalibur ‘automates the test selection depending on the genetic region to be explored’. This is quite unclear and not really developed in the rest of the manuscript.
- The Excalibur.R function that we propose to the readers also return which test within the ensemble function identified a particular genetic region on which the test was performed. It gives an insight to the user on which mathematical test model was able to identify the region. For example, if genes were identified by a test able to handle protective variants (*e.g.* Variance-component tests), the user might consider that the genes carry such variants. We revised the manuscript in order to avoid confusion: “Excalibur also provides a comprehensive elucidation of the underlying genetic architecture pertaining to each genomic region to be investigated.”
- In addition to the proportion of inflated, it would be useful to have the proportion of conservative tests as it seems to be the case for Excalibur.
- We added a column with the proportion of conservative tests and revised the manuscript accordingly.
- l. 284: Please clarify the sentence around the ‘mathematical models’. ID9 represents a scenario with a larger region than scenarios 3 and 8: can the authors explain why no results are obtained?
- Several tests failed to return a p-value on different occasions. When investigating the errors returned, we faced that for some simulations, some tests output an error coming from their mathematical model, or simply a NA p-value. This means that researcher running a particular test for an entire genome might not obtain results (or the entire analysis could crash) for some genes. This discovery is both intriguing and merits further inquiry; however, we underscore that such an exploration lies outside the scope of the present manuscript.
- Table S2 would be useful as a main table as a lot of results refer to it.
- We integrate Table S2 within the manuscript as Table 1.
- Could the authors give more digits in Table S4? This would be useful for the last significance threshold but also for a threshold of 0.01 when results are borderline (as for WGScan).
- We added digits and updated the table, called Table S3 in the revised manuscript. Note that 0,0000 stand for 0 meaning that the test never returned a significant p-value.
- Please define more precisely what is in Table S10 and how the evolution is computed.
- In the original manuscript, evolution is defined at lines 364-365 as “Evolution as the type of change (e.g. increase, decrease, no change, …) in type I error or empirical power when increasing a parameter value”. We improved the description of Table S.10 in the new manuscript. For example in Table S10, the evolution in column I is an interpretation of the values present in column F and G. If we look at line 4 of that Table, we can see that both column F and G are positive, and therefore the evolution is an increase.
- l. 145: what does ‘out-of-the-box’ mean?
- By out-of-the-box we meant tests that failed to return any p-value at step 1.B. We removed it from the manuscript as it seems to generate confusion. These particular implementations of tests taken from the literature did not return any p-value during our preliminary test selection, and where removed (*i.e.* p_assuw, p_assuw_ord, p_wgscan_disp, p_wgscan_burden, p_lasso_multi and p_lasso_cox).
- l. 153: If a function has a wider range of computation time, for example going from 16 to 24 seconds, it will be privileged over others with a lower range but a lower computation time, for example going from 6 to 8 seconds. Should the mean computation time also be considered?
- Based on our equation (see line 153 of the original manuscript) $Max_{evol}=\frac{Max\left( time \right)-Min(time)}{Min(time)}$ , your example would be (24 – 16) / 16 = 0.5 and (8 - 6) / 6 = 0.33. Therefore, the second test will rather be privileged as we take the minimum of Max_evol (to minimize the computational time explosion).
- l. 163: what are the three scenarios considered? The three cohort sizes?
- In this last step of our preliminary test selection, we used our simulation framework using input parameters described in file step_4_input_param_simulation, which can be found in the Github. To overcome the lack of clarity, we added the parameters used within the new Table 1 as preliminary power experiment, and modified the manuscript.
- l. 194: Why the number of causal variants need to be respected for the simulations under the null?
- Most aggregation tests require at least two variants (a genotype matrix with two columns) and can’t handle a single variant scenario since they have been developed to handle several variants. Moreover, when talking about aggregation, we always refer to a situation where several variants are taken into account at the same time, in opposition to single variant testing. This is why in our method we always ensure to have at least two variants.
- l. 254: the proportion should be divided by the number of scenarios for which a type I error was estimated. Similarly in line 298, it should be divided by the number of experiments that gave a p-value.
- Because the ability of a test to control type I error depends on the alpha level at which it has been evaluated, we need to divide the number of alpha level that was evaluated by the number of experiment times. For each test, we give the results (did control well the type I error: Yes or No) for each experiment at each alpha level, as explained in the original manuscript (see lines 252-253). Therefor, to compute the proportion, for type I error, we need to divide by 27. Line 298 refers to another concept, related to last major comment of reviewer 1. We did use that concept for type I error. But, if we compute the power as the number of significant p-values divided by the number of experiments that gave a p-value (as suggested by the reviewer), it would lead to an artificial increase in power for tests with a low reliability (tests that fail to return a p-value). Therefore, we rather divided by the number of replicates (*i.e.* 1000). For example, consider an experiment where both test A and B returned 500 significant p-values. Test A has a reliability of 0.9 and test B of 1, which means test B never failed to return a p-value. Using our definition, both tests will have the same power. If we defined power as suggested by the reviewer, test A will have a greater power of $\frac{500}{900}=56\%$ than test B (50%), disregarding the fact that in 100 repeats, test A failed to return a p-value.
- l. 421: Why can’t this be extended to other alpha levels?
- Looking at Table S10 we can see that in most cases this can be extended to other alpha levels, but not always. For example, we can see an increase in power for Excalibur when increasing the proportion of cases/controls looking at nominal alpha level 0.05 and 0.01, and a decrease at alpha level 0.001. This can be explained, as highlighted by the reviewer, by the type I error and power of a particular test to depend on the alpha level at which it has been evaluated.
- l. 451: It sound like the type I error is computed again, is it really the case or simply the minimal type I error?
- Each time a test is added to the ensemble method, the minimal p-value has to be re-computed, and therefore the type I error as well.
- l. 465: How is the combined rank defined?
- As defined at lines 461-465 in the original manuscript, the combined rank is the sum of the rank and the rank type I. For example, a test with the best score will have a rank of 1, but if that test has the third smallest proportion of inflated type I error in set A, then it will have a rank type I of 3. Its combined rank will be 4. This combined rank therefore takes into account both the power and the type I error performance compared to the other tests in set A.
- Figures S2 and S3: Why p_rvt2 is in the badly corrected and not the goodly corrected panel?
- Because when computing the confidence interval, the upper blue horizontal line is at 0.0544, while the type I error of p_rvt2 at nominal alpha level 0.05 for experiment 4 (see Table S3), is equal to 0.0553.
- l. 227: it is stated that this model is used to generate data under the null hypothesis. I think this is a mistake, especially as the null model is already stated line 199.
- Indeed, we corrected the mistake within the revised manuscript. We meant the alternative model.
- l. 240: Table S4 is cited before Table S3
- We fixed the issue in the revised manuscript.
- l. 261: I think the authors refer to Fig S2 and S3 and not S1 and S2
- Yes, we corrected the mistake within the revised manuscript.
- l. 273: typo error for the upper bound of the confidence interval for alpha = 0.01
- Indeed, the upper bound of the confidence interval for alpha 0.01 is 0.0121 and not 0.121. We corrected it in the new manuscript.
- l. 287: there is an extra ‘s’ in Tables S13
- We corrected it in the revised manuscript.
- Table S11 is not cited in the text and table S12 is not really described.
- There was a mistake in the manuscript. We corrected it at line 431 of the revised manuscript, where Table S11 should be cited. We also improved description of Table S12 in the revised manuscript.

# Reviewer 2

- One issue that needed be clarified is that the authors claimed that “Excalibur also automates the test selection depending on the genetic region to be explored (e.g. genes, pathways, etc).” However, it is not crystal clear from the construction of the methods on page 13 how Excalibur choose the tests depending on the genes or pathways. Some explanations would be helpful to strengthen this claim.
- Reviewer 1 pointed this out as well (see above). Thus, we clarified and revised the manuscript accordingly.
- Also, it would be interesting to see if Exacalibur would also have outstanding performance in the mixed populations with people from different ancestries. The variants effects may differ in size and even in direction in different ancestry populations.
- We agree that it would be an interesting investigation to pursue in the future but it falls beyond the scope of this manuscript. Two very interesting papers tackling this issue can be found at <https://doi.org/10.1371/journal.pone.0207677>. More interestingly, a recent method ([10.1016/j.ajhg.2018.12.012](https://doi.org/10.1016%2Fj.ajhg.2018.12.012)) could be a great starting point to build a simulation framework able to explore more in detail the performance of Excalibur and other methods in mixed population settings.

# Reviewer 3

- Include the robust binary SKAT recently developed (Zhao et al., 2020, AJHG) in the comparison.
- As suggested, we included three new tests in our simulations, namely robust_burden, robust_SKAT and robust_SKATO, taken from Zhao et al., 2020, AJHG and re-run all simulations.
- Include those SKAT versions removed due to computing time to have a direct comparison with the proposed ensemble method on type I error, power, and computing time.
- The reviewer states that “However, the comparison design excludes powerful SKAT tests, which is the state-of-the-art method for rare variant analysis, simply due to strict computation time”. In fact, not all SKAT tests where removed, only the less efficient ones. Out of the 56 state-of-the-art methods under comparison, 10 come from the original SKAT package. Two Burden tests (*i.e.* p_linear_liu_burden and p_linear_weighted_liumod_burden), six forms of the powerfull SKAT (*i.e.* p_linear_davies_skat, p_linear_weighted_davies_skat, pBin_linear_IBS_SKAT_MA, pBin_weighted_IBS_SKAT_MA, pBin_2wayIX_SKAT_MA and pBin_weighted_quadratic_SKAT_ERA) and two SKATO (*i.e.* pBin_linear_SKATO_MA and pBin_linear_weighted_SKATO_MA).
- When comparing power, there is no need to discuss methods that cannot control type I error
- Indeed, we agree and revised the manuscript accordingly.
- Line 473-474: “minimum p-value from the set of tests included in the ensemble method after multiple testing correction using Benjamini-Hochberg”. Is the correction using the Bonferroni correction or Benjamini-Hochberg? The former is commonly used and is robust against correlated test statistics. The latter is used to calculate the false discovery rate (FDR) and in theory it requires independence of test statistics. Whether the FDR adjustment can be used to control type I error in theory is in question.
- We took into account the remark, and used Bonferroni correction instead of Benjamini-Hochberg.
- I appreciate that many comparisons have been done. However, it is important to summarize the results that can help users to decide which tests to choose in which scenarios. For example, for each scenario, identify the top 3-5 methods. All methods with obvious inflated type I errors should not be included, which should be removed at the first place.
- The main purpose of Excalibur is to overcome the challenge of selecting the right statistical tests that researchers deal with. Therefore, we built and propose our ensemble method Excalibur as a method that enables to control type I error in all scenarios, while achieving the best average power across all scenarios that we tested. We change the manuscript and added: In the 18 empirical power experiments, the best three methods are always our three ensemble methods, except for experiments ID n° 10 where robust_SKAT and robust_SKATO are better.
- The main figures are all blurred.
- We changed our Figures and used the PACE program from PLOS to meet the journal requirements for Figures.
