# Supplementary material for: Excalibur: A new ensemble method based on an optimal combination of aggregation tests for rare-variant association testing for sequencing data
Source: PLoS Comput Biol. 2023 Sep 14;19(9):e1011488. doi: 10.1371/journal.pcbi.1011488 (PMC10522036; doi:10.1371/journal.pcbi.1011488)
Supplement: Attachment — Submitted filename: Excalibur_R1_Review.docx [file pcbi.1011488.s028.docx]

# Response to authors’ comments

*To evaluate test similarities, we compared all p-values from each test at nominal alpha 0.05 to establish their similarities (see new Figures S7 and S8). The highest similarity was achieved by p_cast_chisq/p_rvt2. These tests agreed (both significant or both not significant) in 96% of the simulations. The algorithm to construct Excalibur takes a test into account only when it is the only significant test for a simulation, which helps counterbalance the loss of power caused by multiple testing correction. A test will not be added to the ensemble method if it results in a decrease in power. Surprisingly, p_assu/p_assu_ord and p_ssu/ p_ssu_asymptotic only have a similarity of 53% and 46%, respectively.*

I think these figures help in visualizing the correlations between the remaining tests in Excalibur. However, I am still a little bit surprised that such high correlations between the remaining tests exist. This is especially true for p_rvt1 and p_rvt2 which correspond to very similar tests. Can the authors comment on this? Will both tests be included in Excalibur? I think it is not a disadvantage of Excalibur, but the authors could gain from removing this type of highly correlated tests in terms of computation time, but also because I expect that the conservative behavior of Excalibur is partly related to this issue.

*We completely agree with the reviewer. We parallelized the simulation framework (new code is available at https://github.com/dduv-ddit/excalibur_simulations). However, due to computational time concerns, we restricted our simulations to 10 000 repeats for each type I error experiment. In order to evaluate at a genome-wide significance, 10^7 simulations per experiment would be required to expect at least 10^7 * (2.5*10^(-6)) = 25 false positive results. With the computational power available at the institute, it would require a year to perform that many simulations (2 nodes with 16 cores (AMD EPYC MILAN 7313 Processor at 3GHz) and 100 Gb or RAM (DDR4 at 3200 MHz)). Moreover, most aggregation articles do not evaluate type I error at such threshold. Some papers implement different procedures to evaluate such thresholds (*[*https://doi.org/10.1093/biostatistics/kxs014,10.1016/j.ajhg.2011.05.029,10.1016/j.ajhg.2012.06.007*](https://doi.org/10.1093/biostatistics/kxs014,10.1016/j.ajhg.2011.05.029,10.1016/j.ajhg.2012.06.007)*, 10.1016/j.ajhg.2019.11.012). However, they are not described well enough to be reproduced and we do not have access to their supplementary data or code used to perform such simulations. We contacted the authors, without success.*

I understand that running that many simulations to estimate type 1 error requires a lot of resources. However, power could still be estimated at the GW significance threshold as less simulations are needed to assess statistical power than type 1 error.

*In order to answer the question, we changed our experiments with a cohort size of 200 to 5000. Now, the four cohort sizes being investigated are 100, 500, 1000 and 5000. We also added a section to discuss the computational time of each method in the revised manuscript.*

This new section in the manuscript is useful. However, table S13 is not the easiest one to read as it does not explicitly state the parameters used in the simulations (for example the sample size). In addition, the computation time is mostly useful for Excalibur. I would therefore suggest adding a figure showing the computation time according to the sample size for Excalibur rather than this table. The computation time of Excalibur is quite high, which is a limitation of the method. It would be good to at least mention this limitation in the discussion of the manuscript. In addition, the authors mention that a solution would be to parallelize the analyses, is this something already implemented in Excalibur?

*This is based on the fact that sometimes some tests do not manage to return a pvalue (see reliability). Therefore, using the number of significant signals divided by the number of replicates, for example for the test, returned 10 / 10,000 = 0.0010, but if reliability was 0.9, we would only have 10 / 9,000 = 0.0011.*

I totally agree with the authors, but it is not what is stated in the text, l. 366: “Type I error was equal to the number of significant p-values divided by the number of non significant p-values”.

*We added a column with the proportion of conservative tests and revised the manuscript accordingly.*

Just to clarify, do the authors consider conservative type I error to be ‘well-controlled’ type 1 error? If not, Figure 1 would need to be changed accordingly, as well as table S4 where ‘conservative’ is currently a subset of ‘well-controlled’.

# Minor comments

- Please check the typos and phrasing in the whole manuscript (example in lines 312, 348, 380, 408, 509-510, 517).
- Avoid the word ‘patients’ (line 119), and change n_p_ nomenclature accordingly.
- Table S2 is listed before Table S1
- L. 219: there is no ‘Step 5 above’
- L.520-521: “… to analyze 20,000 genetic regions (the entire genome)…”

The analysis of 20,000 regions corresponds to the exome, but not to the entire genome. It is expected that much more tests will be performed when considering the whole genome using WGS data (<https://link.springer.com/article/10.1007/s00439-020-02190-y>, <https://www.nature.com/articles/s41467-019-11023-0>, <https://journals.plos.org/plosgenetics/article?id=10.1371/journal.pgen.1009923>), either by applying sliding window approaches, or pre-defined regions outside the genes.

- Overall, the references are a little bit old, especially in reviewing rare variant association tests, please update using more recent ones such as <https://www.nature.com/articles/s41576-019-0177-4>, <https://www.frontiersin.org/articles/10.3389/fgene.2022.1014947/full>. Similarly, important papers have been left out, including studies aiming at combining multiple rare variant association tests (<https://www.nature.com/articles/ejhg2015194>)
