# Supplementary material for: Excalibur: A new ensemble method based on an optimal combination of aggregation tests for rare-variant association testing for sequencing data
Source: PLoS Comput Biol. 2023 Sep 14;19(9):e1011488. doi: 10.1371/journal.pcbi.1011488 (PMC10522036; doi:10.1371/journal.pcbi.1011488)
Supplement: Attachment — Submitted filename: response_to_reviewers_20230829.docx [file pcbi.1011488.s029.docx]

We would like to express our gratitude to the reviewers for the valuable time and effort dedicated to reviewing our manuscript. Your insightful comments and constructive feedback have been immensely beneficial in improving the quality and clarity of our work.

# **Reviewer 1**

## Major comments

- *Authors previous response: To evaluate test similarities, we compared all p-values from each test at nominal alpha 0.05 to establish their similarities (see new Figures S7 and S8). The highest similarity was achieved by p_cast_chisq/p_rvt2. These tests agreed (both significant or both not significant) in 96% of the simulations. The algorithm to construct Excalibur takes a test into account only when it is the only significant test for a simulation, which helps counterbalance the loss of power caused by multiple testing correction. A test will not be added to the ensemble method if it results in a decrease in power. Surprisingly, p_assu/p_assu_ord and p_ssu/ p_ssu_asymptotic only have a similarity of 53% and 46%, respectively.*
- **I think these figures help in visualizing the correlations between the remaining tests in Excalibur. However, I am still a little bit surprised that such high correlations between the remaining tests exist. This is especially true for p_rvt1 and p_rvt2 which correspond to very similar tests. Can the authors comment on this? Will both tests be included in Excalibur? I think it is not a disadvantage of Excalibur, but the authors could gain from removing this type of highly correlated tests in terms of computation time, but also because I expect that the conservative behavior of Excalibur is partly related to this issue.**
- We have addressed the reviewer's insightful comment in the manuscript's discussion section. Our primary focus has been on ensemble methods that strike a balance between optimizing power and avoiding inflated type I errors. However, it's worth noting that alternative ensemble methods could be designed with different objectives, such as minimizing computational time and reducing correlated tests further, potentially resulting in less conservative but maybe also less powerful approaches.

To explore this concept, we conducted an experiment in which we systematically removed one of the two tests (specifically the one with lower power) within Excalibur for all test pairs exhibiting a similarity greater than 0.8. These tests included p_rvt2, robust_SKATO, p_cast_chisq, p_rwas, p_rebet, and p_wep. The modified Excalibur maintained a consistent balance between conservative behavior and good type I error control, yielding proportions of 0.963 for conservatism and 1 for good type I error. However, we did observe a marginal reduction in statistical power, with the revised Excalibur achieving a power of 0.731 compared to the 0.734 achieved by the Excalibur version outlined in the original manuscript.

As expected, the modified Excalibur version, with six fewer tests, demonstrated improved computational efficiency: In the best scenario (see Table S13), the computational time decreased from 58.01 hours to 52.26 hours, resulting in a noteworthy 9% enhancement in computational speed. In the worst (see Table S13) scenario, the computational time decreased from 458.25 hours to 403.18 hours, corresponding to a substantial 12% gain in efficiency. Our manuscript concentrated on the development of an ensemble method that prioritizes power optimization while avoiding inflated type I error.

In aggregate, we acknowledge the reviewer's suggestion regarding the creation of ensemble methods based on alternative criteria, to ensure reasonable correlation among tests. This avenue of research opens up possibilities for future investigations, potentially leading to ensemble methodologies that cater to various optimization goals beyond power enhancement.

- *Authors previous response: We completely agree with the reviewer. We parallelized the simulation framework (new code is available at https://github.com/dduv-ddit/excalibur_simulations). However, due to computational time concerns, we restricted our simulations to 10 000 repeats for each type I error experiment. In order to evaluate at a genome-wide significance, 10^7 simulations per experiment would be required to expect at least 10^7 * (2.5*10^(-6)) = 25 false positive results. With the computational power available at the institute, it would require a year to perform that many simulations (2 nodes with 16 cores (AMD EPYC MILAN 7313 Processor at 3GHz) and 100 Gb or RAM (DDR4 at 3200 MHz)). Moreover, most aggregation articles do not evaluate type I error at such threshold. Some papers implement different procedures to evaluate such thresholds (*[*https://doi.org/10.1093/biostatistics/kxs014,10.1016/j.ajhg.2011.05.029,10.1016/j.ajhg.2012.06.007*](https://doi.org/10.1093/biostatistics/kxs014,10.1016/j.ajhg.2011.05.029,10.1016/j.ajhg.2012.06.007)*, 10.1016/j.ajhg.2019.11.012). However, they are not described well enough to be reproduced and we do not have access to their supplementary data or code used to perform such simulations. We contacted the authors, without success.*
- **I understand that running that many simulations to estimate type 1 error requires a lot of resources. However, power could still be estimated at the GW significance threshold as less simulations are needed to assess statistical power than type 1 error.**
- Thank you for the suggestion of assessing statistical power at the GW significance threshold.

While we acknowledge that power assessment could be conducted at the GW significance threshold with fewer simulations, our manuscript adheres to a coherent structure established based on previous reviewer feedbacks. The emphasis has been on ensuring the control and validity of type I error before delving into discussions related to power analysis.

Maintaining this sequence of analysis aligns with methodological rigor and logical consistency. By first establishing the foundation of accurate type I error estimation, we then create a robust framework for discussing and comparing the statistical power of various tests. As such, our manuscript refrains from presenting power results at alpha levels without a prior estimation of type I error to sustain the manuscript's overarching consistency and scholar integrity.

- *Authors previous response: In order to answer the question, we changed our experiments with a cohort size of 200 to 5000. Now, the four cohort sizes being investigated are 100, 500, 1000 and 5000. We also added a section to discuss the computational time of each method in the revised manuscript.*
- **This new section in the manuscript is useful. However, table S13 is not the easiest one to read as it does not explicitly state the parameters used in the simulations (for example the sample size). In addition, the computation time is mostly useful for Excalibur. I would therefore suggest adding a figure showing the computation time according to the sample size for Excalibur rather than this table. The computation time of Excalibur is quite high, which is a limitation of the method. It would be good to at least mention this limitation in the discussion of the manuscript. In addition, the authors mention that a solution would be to parallelize the analyses, is this something already implemented in Excalibur?**
- Your feedback is greatly appreciated. We have made improvements to the manuscript accordingly.

Table S13 in the manuscript has been designed to provide an overview of the simulation results, but we acknowledge that it could be more user-friendly in terms of explicitly stating the parameters used in the simulations, including the sample size. To address this, we have included a new figure, Figure S12, which depicts the computation time of Excalibur across various cohort sizes. The legend for Figure S12 is as follows: *"Boxplot illustrating the computational time (in seconds) required by the Excalibur method across varying cohort sizes. The x-axis represents the cohort size, while the y-axis denotes the computational time in seconds. The distribution of computational time is visualized using boxplot, providing insights into the method's efficiency as cohort size changes."*

We now explicitly discuss the computational limitations of Excalibur in the manuscript's discussion section in the following paragraph: “*The main limitation of our ensemble method is that it is conservative and computationally intensive. While our primary focus remained fixed upon constructing an ensemble method that accentuates statistical power, we concede that the avenue of constructing an ensemble method guided by distinct criteria – for example centered around judicious correlation management between tests – warrants further exploration. The trade-off between computational efficiency and statistical power underscores the complex choices that underlie the optimization of ensemble methods. Moreover, we did not explore all the current limitations of aggregation tests, such as incorporating variant annotations to enhance statistical power by assigning functional relevance to variants or addressing the impact of population structure (100), we left such limitations for future exploration”*

Regarding the implementation of parallelization in Excalibur, the method available in R package does not have built-in parallelization argument. However, as the method is a “per-gene” computation, using it in a parallel environment such as an HPC cluster is quite straightforward (*i.e.* submitting 1 job per gene).

- **I totally agree with the authors, but it is not what is stated in the text, l. 366: “Type I error was equal to the number of significant p-values divided by the number of non significant p-values”.**
- Indeed, we corrected the manuscript accordingly. Thank you for pointing out the mistake.
- **Just to clarify, do the authors consider conservative type I error to be ‘well-controlled’ type 1 error? If not, Figure 1 would need to be changed accordingly, as well as table S4 where ‘conservative’ is currently a subset of ‘well-controlled’.**
- Yes, our goal was to separate methods with inflated type I error from the others. Therefore well-controlled also includes conservative methods.

## Minor comments

- **Please check the typos and phrasing in the whole manuscript (example in lines 312, 348, 380, 408, 509-510, 517).**
- We revised the manuscript accordingly.
- **Avoid the word ‘patients’ (line 119), and change np nomenclature accordingly.**
- We changed it to cases and change the nomenclature to Case $n_{cases}$ and control $n_{controls}$.
- **Table S2 is listed before Table S1.**
- We corrected this mistake.
- **L. 219: there is no ‘Step 5 above’.**
- Indeed, it was step 4. We revised the manuscript accordingly.
- **L.520-521: “… to analyze 20,000 genetic regions (the entire genome)…” The analysis of 20,000 regions corresponds to the exome, but not to the entire genome. It is expected that much more tests will be performed when considering the whole genome using WGS data (https://link.springer.com/article/10.1007/s00439-020-02190-y, https://www.nature.com/articles/s41467-019-11023-0, https://journals.plos.org/plosgenetics/article?id=10.1371/journal.pgen.1009923), either by applying sliding window approaches, or pre-defined regions outside the genes?**
- We revised the manuscript, indeed we were talking about the whole exome.
- **Overall, the references are a little bit old, especially in reviewing rare variant association tests, please update using more recent ones such as https://www.nature.com/articles/s41576-019-0177-4, https://www.frontiersin.org/articles/10.3389/fgene.2022.1014947/full. Similarly, important papers have been left out, including studies aiming at combining multiple rare variant association tests (https://www.nature.com/articles/ejhg2015194).**
- We appreciate your valuable input regarding the references. We strive to ensure the inclusion of up-to-date and relevant literature.

We integrated " Rare-variant collapsing analyses for complex traits: guidelines and applications" which is available at (https://www.nature.com/articles/s41576-019-0177-4).

We acknowledge that this review provides a comprehensive overview of the multiple factors that demand attention when utilizing aggregation methods in various contexts.

Regarding the article "Recent advances and challenges of rare variant association analysis in the biobank sequencing era," available at (https://www.frontiersin.org/articles/10.3389/fgene.2022.1014947/full), we acknowledge its relevance and significance, and have successfully integrated this important review into our manuscript's discussion section.

The article titled " A general approach for combining diverse rare variant association tests provides improved robustness across a wider range of genetic architectures" (https://www.nature.com/articles/ejhg2015194) is indeed a noteworthy addition to the discourse. Unfortunately, it appears that the code associated with this study is no longer accessible at the provided link (http://www.dordt.edu/academics/programs/math/statgen/software.shtml). However, we value the significance of this work and incorporated the study's findings into our manuscript.

# Reviewer 2

- **The authors revised the manuscript nicely, and explained investigating Excalibur performance on mixed populations is out-of-scope, which is acceptable. Some comments on line 290: suggest to use "log" instead of "ln" to make it consistent with line 288 where it uses log(MAF). Also suggest to delete "0,402" since the rest cs calculated with different MAFs do not show an associated approximate value. Correct the format for the first c.**
- The use of log at line 288 is in the equation to set the effect size equation $\left| \beta_{j} \right|=c \left| \frac{{log(MAF}_{j})}{2} \right| \forall j \in1, .., m_{c}$, as proposed in (41, 42, 46, 83). But within this equation, the constant “c” is set to c = $\frac{ln(5)}{4}$, $c= \frac{ln(7)}{4}$ and $c= \frac{ln(13)}{4}$ when the percentage of causal variants was 20%, 10% or 5 % respectively, as proposed (41, 42, 46, 82). *We removed the approximate value for the first value of c in the manuscript as suggested*.

# Reviewer 3

- **Table 1 is not complete in the PDF file, so it is hard to check in which scenario which methods are performing well and whether the results make sense.**
- We revised Table 1 so that it fits entirely within the manuscript.
- **The powers of the proposed methods are much higher than the previous version. The previous proposed methods all have power < 0.6. The suggested Robust binary SKAT is already > 0.6 in the new version. Is it due to the inclusion of the suggested robust binary SKAT recently developed (Zhao et al., 2020, AJHG)? Please add comments or discussions in the paper.**
- One contributing factor to the overall enhancement in power is a modification to the power scenario (experiment ID 7) within our analysis. Specifically, we altered the cohort size from 200 individuals to 5000 individuals. As a result, the power of the methods naturally increased due to the larger sample size, leading to improved statistical power across the board.

However, we also acknowledge the significant impact of the new test introduced based on your recommendation. Upon examining Figure 2, it becomes evident that three of our ensemble methods, followed by robust_SKAT and robust_SKATO, occupy the upper right part of the plot. This positioning indicates a good type I error along with higher power, which can be attributed to these methods' ability to detect associations more effectively.

Given that these tests are integral components of our ensemble methods, there is no doubt that their inclusion has significantly contributed to the observed enhancement in our ensemble methods' power.

The manuscript has been updated to reflect this additional insight, ensuring that our readers are informed about the role of robust_SKAT and robust_SKATO methods in boosting the performance of our ensemble approaches. We added in Discussion : *“The two new tests robust_SKAT and robust_SKATO perform better than state-of-the-art methods in most experiments.”*

- **The type I error of the robust binary SKAT/SKATO is 0 when the nominal value is 0.05. This is different from the paper (Zhao et al., 2020, AJHG) which shows that the type I error is very close to the nominal level. Can the authors explain? Is it because the sample size or other settings are different? It will be more convincing if the authors can show that when the simulation setting parameters are changed to that used in (Zhao et al., 2020, AJHG), then the type I error is close to the nominal.**
- We appreciate your observation. This disparity could indeed be attributed to various factors, such as differences in sample size, specific settings, or simulation parameters.

While we acknowledge the potential for differences in simulation settings between our study and Zhao et al.'s work, it is important to note that the exact reproduction of their simulations would require specific parameters and access to their simulation procedures. Unfortunately, these key parameters and simulation setups are not all presented in the article nor available to us so far.

Additionally, attempting to replicate the exact simulation conditions from Zhao et al. (2020) or other state-of-the-art methods considered in our manuscript (reference 59) is beyond the scope of our study. Our simulation framework aims to capture the most recurrent forms and parameters encountered in the literature while acknowledging that not all parameters can be covered due to the broad range of methodologies and techniques.

We strongly believe that establishing a standardized simulation framework that can be accessed and adopted by the scientific community as a whole would be immensely beneficial. Such an approach would facilitate consistent comparisons and evaluations of various methods, preventing the need for unique in-house simulation frameworks for each new method that emerges.

In summary, while it is challenging to precisely reproduce simulations from external studies due to differences in parameters and settings, we emphasize the importance of establishing a shared simulation framework for robust comparisons across methods, which aligns with the overarching goal of promoting transparency and reproducibility in scientific research.

- **Include those SKAT versions removed due to computing time to have a direct comparison with the proposed ensemble method on type I error, power, and computing time. Here is the list to consider because these methods is likely to be more powerful even though more time is needed. pBin_linear_SKATO_ER, pBin_linear_weighted_SKATO_ER, pBin_linear_SKATO_ERA, pBin_linear_weighted_SKATO_ERA, pBin_linear_SKATO_Hybrid, pBin_linear_weighted_SKATO_Hybrid.**
- We appreciate your suggestion. Considering the computational time constraints, it's important to note that Excalibur already faces challenges in this regard (as pointed out by reviewer 1). To maintain manuscript consistency and ensure a fair comparison among all methods, we performed a preliminary test selection process to exclude computationally expensive methods. This decision was based on the need to strike a balance between methodological comprehensiveness and practical feasibility, particularly in light of Excalibur's existing computational time limitations.

To uphold the manuscript's coherence and prevent making exceptions, we believe it's best to adhere to the same selection process for all methods under consideration. Including additional methods could exacerbate the computational time challenges that Excalibur is already encountering. Moreover, reviewer 1 suggested rather to further remove tests that produce highly concordant results to limit the computational burden.

We acknowledge that exploring ensemble methods using different criteria, without discarding potential candidates based on computational time, holds significance. However, this aspect falls beyond the scope of our current manuscript. Our primary focus remains on providing a consistent, comprehensive evaluation of ensemble methods while respecting the computational constraints that Excalibur presents.

- **Besides the proposed methods, it will add more value to the paper to summarize the results to identify the top 3-5 individual methods for each scenario because users may only consider running one individual method instead of an ensemble method.**
- We revised the manuscript under the Section **Exploring some limitations of aggregation tests** in order to describe the best 5 state-of-the-art methods (discarding our ensemble methods) for each scenario. (Pages 14 to 19.)
